# The Application of Selective Hepatic Inflow Vascular Occlusion with Anterior Approach in Liver Resection: Effectiveness in Managing Major Complications and Long-Term Survival

**DOI:** 10.1155/2021/6648663

**Published:** 2021-04-28

**Authors:** Khai Viet Ninh, Nghia Quang Nguyen, Son Hong Trinh, Anh Gia Pham, Thi-Ngoc-Ha Doan

**Affiliations:** ^1^Viet Duc University Hospital, Hanoi, Vietnam; ^2^Hanoi Medical University, Hanoi, Vietnam

## Abstract

**Background:**

Hepatectomy is always a challenge to surgeons and requires an appropriate approach for specific tumors to achieve effective complication management. Selective hepatic pedicle clamping is more considerable strategy when comparing with total hepatic pedicle clamping in the balance between reducing blood loss and transfusion with causing the hepatic parenchyma damages (two main complications affecting liver resection result).

**Objectives:**

In this study, we aim to describe the application of selective hepatic inflow vascular occlusion (SHIVO) and anatomical anterior approach in liver resection and evaluate the results, focusing on intraoperative and postoperative complications.

**Methods:**

We enrolled 72 patients who underwent liver resection with SHIVO at Viet Duc University Hospital in 4-year period (2011-2014) and then followed up all of them until June 2020 (in 52.6 ± 33 months; range, 2-105 months) or up to the time of death. All the patients were diagnosed with primary or secondary liver cancer, and their future remnant liver volume measured on 64-slice CT scan (dm^3^) to body weight (kg) > 0.8% (for right hepatectomy). Perioperative parameters were collected and analyzed.

**Results:**

The average operation time was 196.2 ± 62.2 minutes, and blood loss was 261.4 ± 202.9 ml; total blood transfusion proportion during and after surgery was 16.7%. Complications accounted for 44.5% of patients in which pleural effusion was the most common one (41.7%). There were no liver failure and biliary fistula after surgery. No deaths were recorded during 30 days postoperatively. Average hospital stay was 11.4 ± 3.7 days. Blood transfusions during the operation and major liver resection were the factors significantly affecting the percentage of complications after liver surgery in our study. In the last follow-up evaluation, 44 patients were dead and 28 patients were alive, in which 7 with recurrence and 21 without recurrence. The overall survival rate was 38.9%.

**Conclusion:**

SHIVO in anatomical liver resection is a safe and feasible approach to help resect precisely targeted tumors and manage several complications in liver resection.

## 1. Introduction

Blood loss and blood transfusion during and after operation are essential prognosis factors for outcome in liver resection. One of the effective methods to limit such major complication is applying SHIVO (hemihepatic or segmental hepatic vascular occlusion). In recent years, SHIVO has been confirmed by many authors, usually in their single-kind hepatectomy (major or minor one) study, that it leads to fewer complications than total hepatic pedicle clamping (Pringle maneuver) as it helps reveal clearly the anatomically resected part of liver and reduce ischemia of remnant liver and intestinal congestion [1–3].

To perform SHIVO, appropriate Glissonean pedicle has to be dissected and controlled for clamping. Bismuth and Makuuchi et al., based on principles of Lortat-Jacob and Ton That Tung methods, launched temporary SHIVO for both hepatic portal vein and hepatic artery of right or left Glissonean pedicle (intrafascial extrahepatic hepatic pedicle approach) to prevent from intestinal congestion and total liver ischemia, particularly in the remnant of liver [4, 5]. Besides, Yamamoto et al., Galperin and Karagiulian, and Launois and Jamieson described Glisson capsule which surrounds all hepatic artery, portal hepatic vein, biliary tract going into hepatic parenchyma, and proposed enbloc Glisson's pedicle approach, which is now called extrafascial Glissonean approach [6–8]. Afterwards, there were many researches of liver resection with SHIVO announcing [1, 3, 9–12]. In this research, we would like to present our experience in applying SHIVO, including both techniques: intrafascial and extrafascial approaches for extrahepatic pedicle isolation in all kinds of liver resection, from right/left hepatectomy, sectionectomy to segmentectomy to assess their safety and feasibility.

## 2. Method

### 2.1. Patients

We prospectively enrolled 72 patients who underwent liver resection with selective hepatic vascular occlusion at Viet Duc University Hospital from September 2011 to May 2014. The selection criteria were patients (i) diagnosed with primary or secondary liver cancer, (ii) whose lesions localized on one side of the liver (left or right), and (iii) with future remnant liver volume measured on 64-slice CT scan (dm^3^) to body weight (kg) > 0.8% (for right liver resection). Exclusion criteria were liver resection related to total hepatic pedicle clamping in any step, requires concomitant bowel resection, combines liver and extrahepatic bile duct resection along with hepaticojejunostomy, or hepatic resection with radiofrequency ablation during surgery for other kind of lesions in the liver.

### 2.2. Surgical Technique

#### 2.2.1. Preparing Patients

*Posture*: patients were positioned supine, and their right arm or both arms are perpendicular horizontally to body. The main surgeon and technician are on the right side, and others are on the left.

#### 2.2.2. Surgical Procedure

*Step 1: laparotomy*: performing upper midline, J-shape, or Mercedes incision depends on size and location of lesions. In our experience, upper midline incision should be only applied for minor liver resection in left liver.

*Step 2: abdominal exploration and tumors evaluation*: we palpate and visually evaluate tumors for their number, size, and location; assess type of liver parenchyma (fibrosis, fibrose, or steatosis); evaluate other organs in the abdomen such as stomach, small intestine, colon, and spleen; and create preliminary assessment of whether hepatic pedicle has either adhesion or enlarged lymph nodes.

*Step 3: liver mobilization*: divide round ligament and falciform ligament and expose the anterior surface of suprahepatic inferior vena cava and root of three hepatic veins.

- *Right Liver Mobilization*. Divide right triangular and coronal ligament and mobilize the liver from diaphragm and right adrenal gland. Right liver mobilization depends on the type of liver resection (partial mobilization or total mobilization to the right side of inferior vena cava or total mobilization with partial or total mobilization of para caval liver portion).
- *Left Liver Mobilization*. Divide left triangular and coronal ligament.
- *Hepatic Vein Control*. Depending on types of liver resection, hepatic vein would be dissected and encircled.

**Figure 1:**
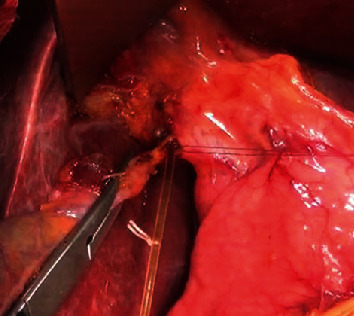
Cholecystectomy and inserting a catheter into main bile duct through cystic duct.

*Step 4: cholecystectomy*: insert a catheter into main bile duct through cystic duct, lymphadenectomy, or not (Figure 1)

- Cholecystectomy is routinely performed except left lateral segmentectomy or segment 2 or 3 resection
- Insert a catheter into main bile duct through cystic duct for bile leakage test, postoperative bile duct drainage, or not
- Lymph nodes of hepatic pedicle (station 12) and common hepatic artery (station 8) would be removed or dissected depending on type of liver tumor, enlarged size of lymph nodes at this step

**Figure 2:**
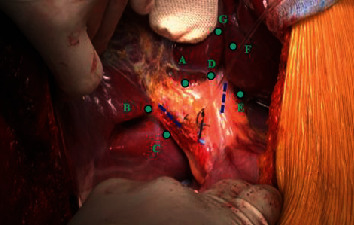
Hepatic pedicle and the site of dissecting for selective Glissonean pedicle control [13, 14].

*Step 5: selective hepatic inflow vascular control for occlusion* (Figure 2)

**Figure 3:**
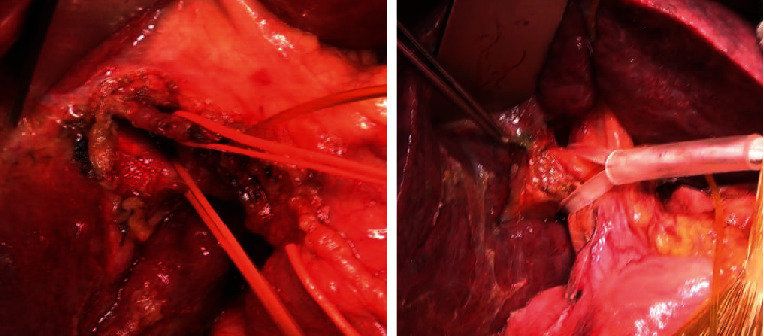
Intrafascial and extrafascial right pedicle approach.

- Right or left Glissonean pedicle–hemihepatic pedicle control
- *Right Glissonean pedicle* (Figure 3)

*Intrafascial right Glissonean pedicle approach*: open peritoneum at upper part of the right side of hepatic pedicle and separately dissect and tape right hepatic artery and right portal vein.

*Extrafascial right Glissonean pedicle approach*: incise Glissonean capsule just above anterior margin of right side of hepatic hilum (point A, Figure 2); incise Glissonean capsule at caudate process, adjacent to posterior margin of hepatic hilum (point C, Figure 2); and apply a curved forceps to dissect two incisions and encircle the right Glissonean pedicle with a tape.

**Figure 4:**
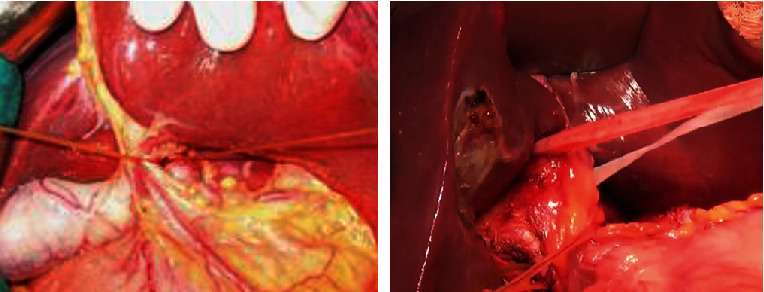
Intrafascial and extrafascial left Glissonean pedicle approach.

- *Left Glissonean pedicle* (Figure 4)

*Intrafascial left Glissonean pedicle approach*: open the peritoneum at the left side of hepatic pedicle, adjacent to hilar fissure bottom (Figure 2), and separately dissect and tape left hepatic artery and left portal vein, noticing to control left accessory or replaced hepatic artery derive from left gastric artery.

*Extrafascial left Glissonean pedicle approach*: incise the Glissonean capsule near the right side of hilar fissure bottom (point D, Figure 2), open the lesser omentum, and perform the second incision adjacent to the posterior margin of hepatic hilum, corresponsive with the first incision (point E, Figure 2). Another second incision could be performed by dividing Arantius ligament adjacent to the lower end of this ligament. Apply a forceps to dissect two incisions for encircling the left Glissonean pedicle with a tape.

#### 2.2.3. Sectional Hepatic Pedicle Control

**Figure 5:**
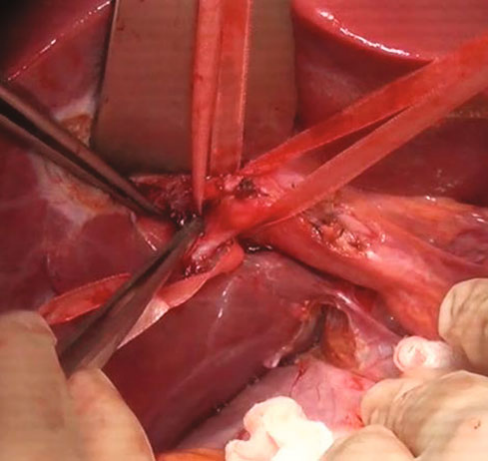
Dissecting right Glissonean pedicle and anterior and posterior Glissonean pedicle.

Sectional hepatic pedicle control was always performed by extrafascial Glissonean pedicle approach at hepatic hilum (Figure 5). The illustration of landmarks and points for dissecting sectional Glissonean pedicle is in Figure 2. For example, anterior segment pedicle is points A and B.

#### 2.2.4. Principles of SHIVO

- *For Right or Left Hepatectomy*. Right or left Glissonean pedicle will be intrafascially or extrafascially approached and clamped continuously.
- For sectionectomy or segmentectomy, the right or left Glissonean pedicle would be dissected as above. Sectional Glissonean pedicle is extrafascially dissected (the main purpose is to realize the transection line based on demarcation). The right or left hepatic pedicle is intermittently clamped every 15-20 minutes during transecting hepatic parenchyma and then released for 5 minutes. The sectional Glissonean pedicle is clamped instead of right or left Glissonean pedicle if the transection line is in the same ischemia area.

*Step 6: transect liver parenchyma*: after approaching and controlling the Glissonean pedicle (either intrafascially or extrafascially), we transected parenchyma basing on the demarcation line and anatomical landmarks [15]. We finally cut the dissected pedicle, which was then clearly exposed. This helped to resect liver more accurately, especially with difficult area such as certain hepatic sections or segments, and avoid damaging to the biliary tract of the remnant liver, particularly in the event of anatomical variation. Therefore, this approach was supposed to reduce ischemia, blood loss, and several complications during and after surgery. The details of these steps are presented as follows.

**Figure 6:**
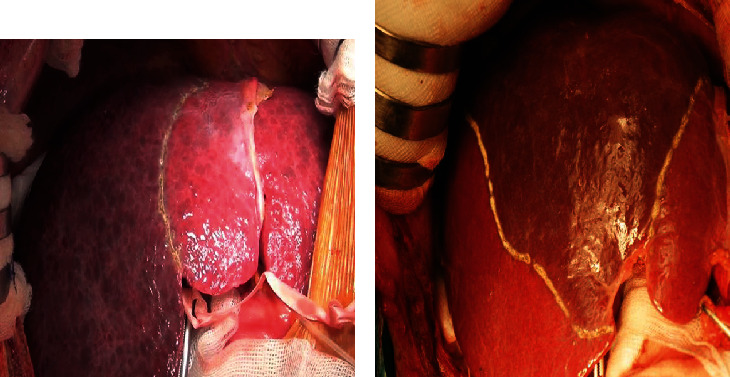
(a) Transection line of right liver; (b) transection line of anterior section.

**Figure 7:**
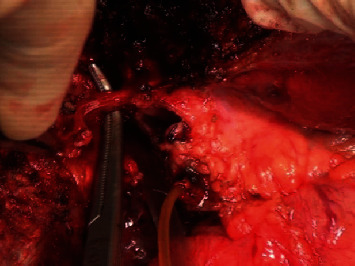
Exposing intrahepatic right bile duct after right portal vein, right hepatic artery ligation, and division and transecting hepatic parenchyma.

- Determine the transection line or plane based on the anatomical landmarks and fissure (Figure 6). If the Glissonean pedicle of removed part of liver is selectively occlusion, then transection line is determined by the demarcation line. We consider a major hepatic resection as resection of three or more segments; otherwise, it is a minor one.
- Resect hepatic parenchyma with Kelly forceps, harmonic scalpel, or CUSA with or without bipolar electrocoagulation while implementing selective occlusion as described above. Intrahepatic vessels were ligated, clipped, and then divided
- Liver parenchyma is transected, and Glissonean pedicle of resected parenchyma is clearly visible in hepatic parenchyma. Ligate or suture and divide Glissonean pedicle within parenchyma (Figure 7).

In the case of right or left liver resection with right or left portal vein thrombosis, hepatic artery and portal vein would be dissected and divided extrahepatically before transecting parenchyma. The biliary tract is eventually divided intrahepatically after parenchyma transection in order to prevent damage to the remnant bile duct due to anatomic biliary variation.

*Step 7: check bleeding and bile leakage and cover transection surface*

- *Check Bleeding*. This is a coagulation with bipolar electrocautery, suturing at oozing sites or ligating the stumps of vessels on transection surface of remnant liver.
- Inject normal saline into the catheter inserted into main bile duct through cystic duct for bile leakage detection and suturing bile leakage sites (if any)
- Cover transection surface with Surgicel, greater omentum, etc.

**Figure 8:**
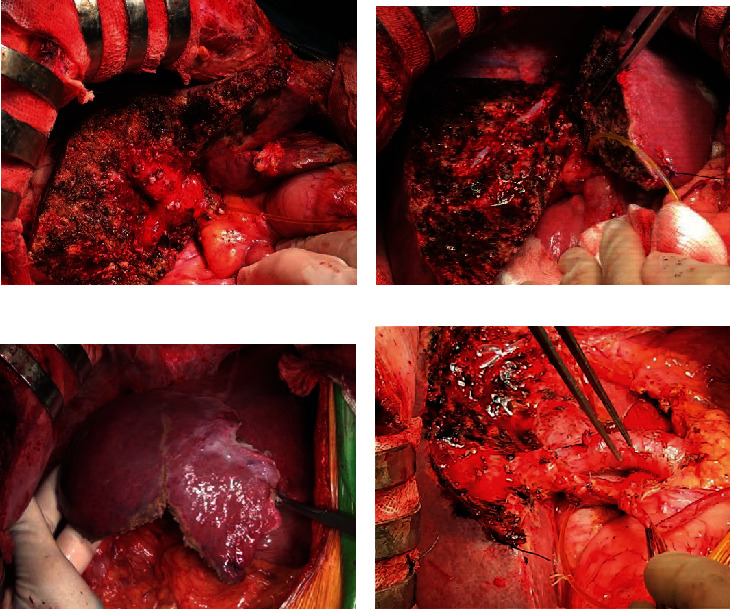
(a) Segment 5 and 6 resection; (b) anterior sectionectomy; (c) segment 5, 6, and 7 resection; (d) left liver plus segment 1 resection and lymphadenectomy.

*Step 8: place drainage and close abdomen* (Figure 8)

### 2.3. Studied Criteria

The studied criteria were as follows: duration of pedicle isolation and overall surgery, blood loss, and transfusion requirements (both intra- and postoperation). Complete blood count and serum biochemistry including renal function and liver function were evaluated. Postoperative morbidity, mortality, and hospital stay were also obtained. Postoperative hemorrhage is defined as considerable blood loss from drainage, hematoma, or blood fluid or active hemorrhage detected by ultrasound or CT scan, and hemoglobin level is decreased 3 g/dl compared with immediately postoperative level. Bile leakage is diagnosed when drain fluid has bile-like color and total bilirubin concentration of drain fluid is more than 5 mg/ml or three times the serum concentration. Ascites is drain fluid amount more than 500 ml/day for at least 3 days or abdominal fluid is considerable in ultrasound. Postoperative liver insufficiency was defined as prothrombin < 50% and bilirubin > 50 *μ*mol on the postoperative day 5. Pleural effusion is defined as more than 3 cm thick fluid of thoracic cavity in ultrasound (supine position). Values are presented as mean ± SD (range) or median (range). Postoperative complications were classified according to Dindo-Clavien [6].

For long-term outcome, all patients were reexamined 1 month after discharge and then followed up every 3 months with blood biochemistry (liver function), tumor markers, and abdominal ultrasound. Abdominal computed tomography (CT) scans were indicated every 6 months or when tumor recurrence is suspected.

During postoperative follow-up, patients with recurrence would be informed and explained for further treatment. The recurrent treatment methods for patients should be the following: TACE, radiofrequency ablation, resurgery, and symptomatic treatment. The last follow-up evaluation was in June 2020 or up to the time of death.

### 2.4. Statistical Analysis

Descriptive data were presented as means and standard deviations (SDs) and percentages. All data were analyzed with SPSS 21.0 (SPSS Inc., Chicago, IL, USA). Using Fisher's exact test, with a *p* value of <0.05 was considered statistically significant.

### 2.5. Ethics

All clinical investigations were conducted according to the principles expressed in the Declaration of Helsinki. Vietnam Military Medical University Institutional Review Board Committee approved the study protocol, and patients provided informed consent before participation. All patients provided written informed consent.

## 3. Results

### 3.1. Preoperative Characteristics

From September 2011 to May 2014, a total of 72 patients were enrolled and received liver resection with SHIVO. Their mean age was 52.3 ± 10.7 years old (range, 23-70 years old). Most patients were men (83.3%). On the past medical history, 77.8% of patients had hepatitis B and/or C, 23.6% had previous TACE plus PVE, and 5.6% had previous single TACE. All patients had Child-Pugh A. There were 62 ones diagnosed with HCC (86.2%), while cholangiocarcinoma and colorectal liver metastasis had an equal proportion (6.9%). The mean tumor size measured by CT scan or/and MRI is 6.7 ± 2.9 cm (range, 1.9–13.4 cm), with 56.9% of patients having tumors > 5 cm and 4.2% of patients had >3 tumors. There were 7 patients (9.7%) figured out with portal vein thrombosis by CT and/or MRI (Table 1).

### 3.2. Intraoperative Parameters

The approaches of Glissonean pedicle were described in Table 2. Intrafascial approach for right/left Glissonean pedicle was achieved in 42 patients (58.3%). Extrafascial approach for right/left Glissonean pedicle accounted for 18.1%. There were 23.6% of patients with extrafascial approach for right/left Glissonean pedicle and sectional/segmental Glissonean pedicle.

In intrafascial approach group, there were 15 patients with previous PVE and 7 ones with portal vein thrombosis. Of these, there were 2 patients with left thrombosis invasive into portal trunk which required dissection of both portal trunk and the right portal vein to prevent the thrombosis from moving to the right portal vein.

While dissecting Glissonean pedicle, bile duct injury occurred in 1 patient with the rate of 1.4%, bleeding was observed in 3 patients (4.2%), of which 2 patients already had parenchymal inflammation due to several previous TACE, both of incidents belonged to extrafascial approach. No patient had portal vein or hepatic artery injury during selective hepatic inflow vascular dissection with both intrafascial and extrafascial approaches.

The average Glissonean pedicle dissection time was 12.5 ± 7.2 minutes, (range, 5-45 minutes). There were 43 patients (59.7%) receiving minor hepatic resection. Cholecystectomy was performed for 61 patients (86.1%), and catheter was inserted into main bile duct through cystic duct for bile leakage checking 49 patients (68.1%), intraoperatively detected bile leakage through injecting normal saline into catheter 18/49 patients and postoperatively keeping the catheter 10/49 patients.

The average hepatic parenchymal transection time and surgical time were 39.9 ± 13.3 minutes and 196.2 ± 62.2 minutes, respectively. The longest procedures belonged to resection of left liver along with segment 1, which was, respectively, 51.7 ± 5.8 minutes and 315 ± 39.7 minutes. The mean blood loss during surgery of the entire study was 261.4 ± 202.9 ml (range 30-1100 ml). Left liver resection had the largest average blood loss with the mean of 436.3 ± 319.4 ml (Table 3).

There were 7 patients having blood loss > 500 ml among 10 patients (13.9%) receiving intraoperative blood transfusion, and all these 7 patients were performed major liver resection and intrafascial Glissonean pedicle approach (3 patients with PVE and 3 patients with portal vein thrombosis). Intraoperative blood transfusion 10 patients accounted for 13.9%, and 7 out of 10 patients were major liver resection. Intra and/or postoperative blood transfusion 12 patients accounted for 16.7%. Margin distance < 0.5 cm accounted for 12.5% (9 patients), 0.5–1 cm accounted for 41.7% (30 patients), and >1 cm is 45.8% (33 patients). That means almost all cases (87.5%) had upper 0.5 cm length from tumor to resection margin. Three patients (4.2%) had lymph node metastasis in 8 and/or 12 station.

#### 3.2.1. Early Postoperative Outcome

*(1) Postoperative Kidney and Liver Function Test*. Renal function of all patients after surgery on days 1, 3, and 5 was completely normal. There was no statistically significant difference (*p* > 0.05) between total bilirubin, albumin preoperative day, and postoperative days 1, 3, and 5. Liver enzymes GOT and GPT increased after surgery, of which the highest level was on day 1 and decreased gradually in the following days, statistically significant difference. On the fifth day after surgery, GOT and GPT reached an average of 60.6 ± 74.8 U/l and 88.9 ± 75.8 U/l, respectively. No major disorders observed in postoperative liver function tests (Table 4).

#### 3.2.2. Complications and Deaths after Surgery

Pleural effusion (41.7%) was the most common complication in the study. Postoperative ascites and bleeding accounted for 22.2% and 1.4%, respectively. No cases suffered from liver failure, bile leakage, or infected incision. No patients died in 1 month after surgery. According to Dindo-Clavien's classification of surgical complications, there were 55.5% of patients with no complications, and grade 1 complications accounted for 37.5%. We also observed no complications of grade 3B-grade 5 after surgery (Table 5).

The average hospitalization time of all patients was 11.4 ± 3.7 days (range 7-29 days).

### 3.3. Factors Affecting the Early Postoperative Outcome

The univariate analysis was used with preoperative and intraoperative data. The statistically significant difference in patients with complication after surgery (based on Dindo-Clavien's classification) was found in the group with blood transfusion (intra- and/or postsurgery), removal or dissection of lymph node 8 and 12, and in major liver resection (*p* < 0.05) (Table 6).

## 4. Long-Term Outcome

The median follow-up was 52.6 ± 33 months (range, 2-105 months). All 72 patients were fully followed. 44 patients have died, and 28 patients were alive: 7 patients with recurrence and 21 without recurrence. The 6-year overall survival rate was 38.9%. In HCC group (*n* = 62), 19 patients were alive, in which 7 patients are with recurrence. This group had disease-free survival rates at 1-, 2-, 3-, 4-, and 5-year of 67.7%, 51.6%, 43.5%, 40.3%, and 37%, respectively. The 1-, 2-, 3-, 4-, and 5-year overall survival rates were 80.6%, 72.6%, 62.9%, 56.5%, and 50%, respectively. In cholangiocarcinoma group, 2 of 5 patients were alive and without recurrence, and 3 patients have died and had survival time of 6, 11, and 52 months. In colorectal liver metastasis group, all 5 patients were dead, and the survival time were 20, 27, 35, 56, and 56 months.

## 5. Discussion

Blood loss and blood transfusion during and after operation are essential prognosis outcomes in liver resection. Therefore, many authors have been proposed vascular control methods to help reduce blood loss in liver resection. In 1908, Pringle first time performed total inflow occlusion (clamping of total hepatic pedicle) in order to decrease blood loss [16]. However, this maneuver caused total hepatic parenchyma ischemia and intestinal congestion. The damage of parenchyma increased in longer pedicle clamping time, especially in patients with chronic liver diseases. Bismuth and Makuuchi et al., based on principles of Lortat-Jacob and Ton That Tung methods, launched temporary SHIVO for both hepatic portal vein and hepatic artery of right or left Glissonean pedicle (intrafascial extrahepatic hepatic pedicle approach) to prevent from intestinal congestion and total liver ischemia, particularly in the remnant of liver [4, 5]. Besides, Yamamoto et al., Galperin and Karagiulian, and Launois and Jamieson described Glisson capsule which surrounds all hepatic artery, portal hepatic vein, biliary tract going through into hepatic parenchyma, and proposed enbloc Glissonean pedicle approach, which is now called extrafascial Glissonean approach [6–8]. Afterwards, there were many researches of liver resection with SHIVO announcing such as Takenaka (1996), Malassagne et al., Wu et al., and more recently Tanaka et al., Fu (2011), and Ji et al. [2, 3, 9–12]. This method not only reduces blood loss in liver resection but also helps to recognize exactly hepatic transection lines based on the ischemic and nonischemic region (demarcation line) in performing selective hepatic inflow vascular control and occlusion. In this paper, we would like to share our experience in applying the SHIVO in liver resection and evaluate the results. We approached the Glissonean pedicles in two ways: extrafascial extrahepatic and intrafascial extrahepatic Glissonean pedicle clamping (Table 2). Among 30 patients with extrafascial dissection (“enbloc technique”), there were 13 patients (18.1%) received right or left Glisson pedicle dissection and 17 patients with right/left Glissonean pedicle and sectional/segmental Glissonean pedicle (23.6%). Intrafascial approach for right/left Glissonean pedicle (right/left portal vein and hepatic artery was separately dissected) was performed in 42 patients, accounting for 58.3%. In our experience, for major liver resection, we have to perform intrafascial approach for Glissonean pedicle in patients with portal vein thrombosis, tumor adjacent, or invasive to Glissonean pedicle. This was to ensure the extensive removal of thrombosis and related portal part as well as invasive tissue surrounding the portal triad and lymph node dissection as well. There were 7 cases of portal vein thrombosis with 2 left-side ones spreading to the portal main trunk that required dissection of portal main trunk and both right and left portal vein branch to prevent the thrombosis moving to the portal vein branch of remnant part of liver. Among 17 PVE patients, there were 2 cases having extrafascial approach and 15 cases having intrafascial approach for right Glissonean pedicle. We prefer to do intrafascial approach in these cases to get rid of pushing embolic material from right portal vein into the left side as if performing extrafascial approach. For other major liver resection, we prefer extrafascial approach due to saving time. Average dissection time of extrafascial approach for right/left Glissonean pedicle was 8.6 ± 2.5 minutes, statistically significantly shorter than intrafascial approach—12.6 ± 5.5 minutes. For minor liver resection, we also prefer performing extrafascial approach for resected sections/segments' pedicle and corresponding right/left Glissonean pedicle.

In terms of Glissonean pedicle division and ligation, there are two points of view. One is performing Glissonean pedicle dissection, and always divide and ligate the Glissonean pedicle before transecting parenchyma, using an encircling tape for counter traction to avoid damage to the remnant Glissonean pedicle [17–20]. However, the others are concern about anatomical variation and the risk of remnant bile duct damage (specially for major liver resection) so the bile duct or Glissonean pedicle was always transected after parenchymal transection [9, 21–23]. This is in line with our study, as we always dissect Glissonean pedicle first (either intrafascial or extrafascial approach) to control and occlude the inflow and then transected liver parenchyma before eventually dividing Glissonean pedicle for either right/left liver or sectional resection. This makes us more confident than transecting the related pedicle right after finding them as the first opinion, based on the 2 following facts. First, when dividing Glissonean pedicle intrahepatically, the anatomical variation of the biliary tract will be more controlled and thus bile duct injury of remnant liver would be limited. Second, the pedicle clearly exposed after parenchyma transection will create a good space for surgeon's manipulation, while the longer resected pedicle length will help pedicle ligation be safer and easier.

While performing this dissection technique, we caused 1 bile fistula (1.4%) and 3 bleeding (4.2%). The bile fistula in the right hepatic bile duct was early discovered and fully sutured in the operation. Bleeding happened in extrafascial approach, from hepatic parenchyma around pedicle. These 2 cases already had previous inflammation due to TACE. We all well handled the three incidents with bipolar electrocautery and light pressure with gauze (Table 2). Mouly et al. reported 6% of biliary fistula and 3% of bleeding in Glissonean approach during right hepatectomy [20]. Similar to us, these incidents are mild and well controlled. When performing the extrafascial approach, we incised the Glisson's capsule near hilar plate with fine line, slightly detach and dissect the Glissonean pedicle from hepatic parenchyma and try to minimally destruct the hepatic parenchyma. Recently, Yamamoto et al. and Sugioka et al. mentioned about Laennec's capsule structure between hepatic parenchyma and Glisson's capsule. The Laennec's capsule covers the entire of liver surface and intrahepatic parenchyma surrounding the Glissonean branches and was observed as a dense fibrous layer with Azan Mallory staining in the histology [24]. Based on this, the dissection of segmental Glisson's pedicle following Takasaki's method is more convenient and safer [24, 25].

When performing anatomical liver resection, it is important to determine the transection line. In our study, this was produced by 2 methods. First is Glissonean pedicle occlusion of resected part of liver that revealed the demarcation line. This method is useful for posterior, anterior sectionectomy, or segmentectomy of right liver that is difficult to determine exact transection line. Second, we also transected parenchyma based on hepatic landmarks and fissures described by Ton That Tung but do not need to dissect and occlude the Glissonean pedicle of resected part of liver such as lateral segmentectomy or segment 4 resection [15]. During transecting hepatic parenchyma, all patients were performed right or left Glissonean pedicle occlusion as principles described in protocol.

Blood biochemical test and coagulation tests were performed on postop days 1, 3, and 5 to monitor liver and kidney function. Table 4 shows that patients after liver resection had completely normal kidney function (urea; creatinine on days 1, 3, and 5 completely in normal range). For liver function, GOT and GPT average after surgery on day 1, 3, and 5 were higher than preoperative day (*p* < 0.05), highest on day 1, and gradually decreased on the following tested days. Average postoperative total bilirubin on the same postoperative days increased compared to preoperative ones (*p* < 0.05), and the highest value is on day 3 before slipping down in day 5. Albumin and prothrombin are lower than before operation (*p* < 0.05) and dropped significantly on the 3^rd^ day. Fluctuation of the above biochemical and coagulation indicators is similar to those of Fu et al. [2]. Fu et al. found that postoperative GOT, GPT, and total bilirubin were lower and significantly decreased faster in selective clamping group compared to complete clamping one, but there are no differences in albumin and prothrombin [2].

Liver surgery is still a challenge for surgeons. Recent studies show that although the postoperative mortality rate is reduced and usually less than 5%, the complication rate is still high. Belghiti et al.'s study of 747 cases of liver resection showed that the postoperative death rate was 4.4%, and the group of diseased liver had significantly higher death rate (9.5%) than the group of normal liver (1%) [26]. The postoperative mortality is 0% for cases of minor liver resection or liver resection due to benign tumors in normal liver group and postoperative complication rate 22% only for liver resection with normal liver group. Jarnagin et al.'s study on 1803 cases of liver cut showed that the postoperative mortality rate was 3.1% but the rate of complications after surgery was 45%. Mortality and complications tend to increase when the volume of hepatic parenchyma resection increases, particularly the mortality and complications up to 7.8% and 75% when resecting up to 6 lower liver segments [27].

In our study, no patients died within 30 days after surgery. Based on Dindo's complication classification, Table 5 shows that 32 (44.5%) patients had postoperative complications, of which the main complications are grade 1 (37.5%). Complications at grade 3A with 2 patients accounted for 2.8%, but both complications were pleural effusion causing respiratory failure needed pleural fluid aspiration. In the study, we did not experience complications of grade 3B to 5. Specifically, Table 5 shows that pleural effusion is the most common complication, accounting for 41.7%. Most pleural effusion is mild or moderate without intervention needed. The percentage of pleural effusion after liver resection is 9.6%-47% in previous studies, and division of the triangular ligament and coronary ligament, blood loss, and cirrhosis are causes of this issue [28]. Ascites and residual fluid collection adjacent to resection edge accounted for 22.2% and 9.7%, respectively. Patients with these complications were stable with medical treatment.

There are many risk factors for complications and mortality after a liver surgery. These factors can be the patient's characteristics (older age, cardiovascular and respiratory diseases, etc.), liver function, tumor's characteristics (location, size, and number), and particularly operative factors related (amount of blood loss, complications during surgery, and level of liver transection). Table 6 showed univariate analysis of factors creating significant difference. In our study, removal or dissection of lymph node 8, 12, intraoperative and/or postoperative blood transfusion, and major liver resection were factors that increased the rate of postoperative complications (*p* < 0.05).

There are different perceptions of factors that influence early results. Kamiyama et al. suggested that surgery time > 360 minutes, blood loss > 400 ml, and blood albumin < 35 g/l are factors leading to complications after liver surgery [29]. For cirrhotic patients, Capussotti et al. considered that the age < 70 years, Child-Pugh liver function B and C, blood or plasma transfusion, total hepatic pedicle occlusion time > 40 minutes, and the number of tumors > 2 are significant risk factors leading to complications after liver surgery [30]. According to Belghiti et al., the degree of liver resection (major or minor) and simultaneous operation on other organs are factors that influence early outcome [26]. Platelets < 100 G/l and blood transfusion during and after surgery according to Taketomi et al. are two factors related to complications after liver surgery [31]. Several studies have testified that surgical resection could be performed safely and led to long-term survival particularly in HCC patients. In the present study, the 1-, 3-, and 5-year overall survival (OS) rates were 89.0, 64.3, and 53.0%, respectively, which is in accordance with previous reports.

To sum up, when performing liver resection with anterior approach in combination with selective hepatic pedicle occlusion (either intrafascial or extrafascial approach), we found that this is a safety procedure without long duration (mean dissection time was 12.5 ± 7.2 minutes) and there were nearly no major complications. Only minor ones like biliary fistula or parenchymal bleeding around hepatic pedicle were observed, which were well controlled.

Bleeding control in this approach is as effective as total hepatic pedicle occlusion when the resected part of liver is completely corresponded with the dissected pedicle [1, 11]. For example, the posterior section was resected while right Glissonean pedicle was occluded or resection of segment 2, 3 or the left lateral segment with left Glissonean pedicle occlusion. This technique also did not cause intestinal congestion and ischemia in the remnant liver (right liver resection with right the Glissonean occlusion would not cause ischemia in the left one). Besides, the ischemia region appeared by the selective hepatic pedicle vascular occlusion (hemihepatic, sectional) also helps determine the exact transection plane, especially when it comes to difficult anterior or posterior sectionectomy procedures. Therefore, the amount of blood loss and blood transfusion in our operation is relatively low. The effectiveness of selective hepatic pedicle vascular occlusion has been also recognized by many authors [1–3, 9].

Bleeding is still happening from the hepatic veins and transection surface of remnant liver during right or left liver resection. However, reducing central venous pressure < 5 cm H_2_O would help reduce blood loss from the hepatic veins [32]. Moreover, with the advances of surgical instruments like CUSA, harmonic scalpel, and ligature, bleeding could be effectively and safely managed. In order to decrease blood loss and optimize remnant liver function especially for hepatic resection for liver diseases, we prefer to perform liver resection with SHIVO with our protocol mentioned above.

## 6. Conclusion

Selective pedicle clamping and anterior approach is a safe, feasible, and effective method to resect precisely targeted tumors in hepatectomy and reduce blood loss, transfusions, and several complications in the remnant liver. While extrafascial approach had shorter dissection duration, intrafascial approach should be recommended in patients with previous thrombosis and portal vein embolization.

## Figures and Tables

**Table 1:** Preoperative characteristics of participants.

Characteristic	Finding
Mean age	52.3 ± 10.7 (23-70)
Male sex—no. (%)	60 (83.3)
Past medical history—no. (%)	
TACE^∗^	4 (5.6)
TACE+PVE^∗∗^	17 (23.6)
Hepatitis B and/or hepatitis C	56 (77.8)
Child-Pugh A—no. (%)	72 (100)
MELD score	6.4 ± 0.9 (min:6, max:10)
Definitive diagnosis—no. (%)	
Hepatocellular carcinoma (HCC)	62 (86.2)
AFP > 200 ng/ml	20/62
Cholangiocarcinoma	5 (6.9)
CA19 − 9 > 37 U/ml	3/5
Colorectal liver metastasis	5 (6.9)
CA19 − 9 > 37 U/ml	1/5
Tumor on CT or/and MRI	
Tumor size (cm)	6.7 ± 2.9 (1.9–13.4)
Tumor size > 5 cm—no. (%)	41 (56.9)
Tumor number > 3—no. (%)	3 (4.2)
Portal vein thrombosis on MRI/CT—no. (%)	7 (9.7)

^∗^TACE: transarterial chemoembolization; ^∗∗^ PVE: portal vein embolization.

**Table 2:** Perioperative characteristics.

Characteristic	Finding
Liver resection type—no. (%)	
Minor hepatic resection	43 (59.7%)
Major hepatic resection	39 (40.3%)
Cholecystectomy – No. (%)	61 (86.1%)
Catheter inserted into main bile duct through cystic duct for biliary leakage check	49 (68.1%)
Intraoperatively detected bile leakage	18/49
Postoperatively keeping the catheter for bile drainage	10/49
Glissonean approach—no. (%)	
Intrafascial approach for right/left Glissonean pedicle	42 (58.3%)
PVE	15 (35.7%)
Portal vein thrombosis	7 (16.7%)
Extrafascial approach for right/left Glissonean pedicle	13 (18.1%)
PVE	2 (2.8%)
Extrafascial approach for right/left Glissonean pedicle and then sectional/segmental Glissonean pedicle	17 (23.6%)
Complications in the Glissonean approach—no. (%)	
Bile duct injury	1 (1.4%)
Bleeding	3 (4.2%)
Pedicle dissection time (minutes)	Mean, range
All patients	12.5 ± 7.2 (5-45)
Intrafascial approach for right/left Glissonean pedicle	12.6 ± 5.5 (7–38)
Extrafascial approach for right/left Glissonean pedicle	8.6 ± 2.5 (5–12)
Extrafascial approach for right/left Glissonean pedicle and sectional/segmental Glissonean pedicle	14.9 ± 11.3 (5–45)

**Table 3:** Parenchymal transection, operation time, and the amount of blood loss during surgery.

	Resected position	Patient—no. (%)	Parenchymal transection time (minutes)	Operation time (minutes)	Amount of blood loss (ml)
Major liver resection	Right liver	16 (22.2)	47.4 ± 11.2	217.2 ± 43.4	416.9 ± 194.8
	Left liver	8 (11.1)	48.6 ± 13.7	219.4 ± 50.4	436.3 ± 319.4
	Left liver+segment 1	3 (4.2)	51.7 ± 5.8	315 ± 39.7	263.3 ± 140.1
	Posterior section+segment 5	1 (1.4)	40	170	100
	Anterior section+segment 6	1 (1.4)	58	270	320
	All major liver resection	29	48.3 ± 11.1	228.1 ± 53.3	392.1 ± 230.5
Minor liver resection	Posterior section	14 (19.4)	41.1 ± 7.9	197.8 ± 46.6	228.6 ± 144
	Anterior section	1 (1.4)	75	210	210
	Segment 5, 6	8 (11.1)	35.9 ± 7.6	186.3 ± 30.2	222.5 ± 100.5
	Lateral section	8 (11.1)	24.1 ± 6.9	122.5 ± 33.3	91.3 ± 27
	Segment 1	3 (4.2)	24.7 ± 5.5	226.7 ± 109.7	116.7 ± 57.7
	Segment 8	2 (2.8)	32 ± 4.2	180	265.0 ± 233.3
	Others	7 (9.7)	29.3 ± 8.4	129.3 ± 37.5	92.8 ± 41.9
	All minor liver resection	43	34.3 ± 11.6	174.8 ± 59	173.3 ± 120.2
All liver resections		72	39.9 ± 13.3	196.2 ± 62.2	261.4 ± 202.9 30-1100

**Table 4:** Biochemical and coagulation indices 1, 3, and 5 days after surgery.

Test	Day 1	Day 3	Day 5
Urea (mmol/l)	4.5 ± 1.7	4.8 ± 1.7	6.4 ± 7.4
Creatinine (*μ*mol/l)	78.1 ± 27	63.8 ± 15.7	66.6 ± 14.9
GOT (U/l)	304.2 ± 205.5	117 ± 113.3	60.6 ± 74.8
GPT (U/l)	278.3 ± 187	190.6 ± 158.2	88.9 ± 75.8
Total bilirubin (mmol/l)	20.3 ± 13.7	22.4 ± 17.1	20.2 ± 20.3
Albumin (g/l)	31.8 ± 4.5	30.7 ± 3.9	32.8 ± 4.7
Prothrombin (%)	74.4 ± 16	72 ± 15.5	79.1 ± 16.9

**Table 5:** Complications and death after surgery.

Complications and death	Patient no. (%)
Pleural effusion	30 (41.7)
Ascites	16 (22.2)
Residual fluid collection	7 (9.7)
Bleeding	1 (1.4)
Sepsis	1 (1.4)
Liver failure	0
Bile leak	0
Effusion	0
Incision infection	0
Deaths	0
Clavien-Dindo's classification of complications after surgery	
Degree—no. (%)	
Grade 0	40 (55.5)
Grade 1	27 (37.5)
Grade 2	3 (4.2)
Grade 3A	2 (2.8)
Grade 3B–Grade 5	0

**Table 6:** Factors affect early postoperative complications.

Factors		Complication		*p*
		No	Yes	
Age	<60 (*n* = 50)	24 (48%)	26 (52%)	*p* > 0.05
	≥60 (*n* = 22)	16 (72.7%)	6 (27.3%)	
BMI	<25 (*n* = 60)	31 (51.7%)	29 (48.3%)	*p* ^∗^ > 0.05
	≥25 (*n* = 12)	9 (75%)	3 (25%)	
Total bilirubin (mmol/l)	<20 (*n* = 63)	34 (54%)	29 (46%)	*p* ^∗^ > 0.05
	≥20 (*n* = 9)	6 (66.7%)	3 (33.3%)	
Removal or dissection of lymph node 8, 12	No (*n* = 37)	26 (70.3%)	11 (29.7%)	*p* < 0.05
	Yes (*n* = 35)	14 (40%)	21 (60%)	
Intraoperative blood transfusion	No (*n* = 62)	38 (61.3%)	24 (38.7%)	*p* ^∗^ < 0.05
	Yes (*n* = 10)	2 (20%)	8 (80%)	
Postoperative blood transfusion	No (*n* = 66)	39 (59%)	27 (41%)	*p* ^∗^ < 0.05
	Yes (*n* = 6)	1 (16.7%)	5 (83.3%)	
Intra and/or postoperative transfusion	No (*n* = 60)	37 (61.7%)	23 (38.3%)	*p* < 0.05
	Yes (*n* = 12)	3 (25%)	9 (75%)	
Resected volume	Minor (*n* = 43)	30 (69.8%)	13 (30.2%)	*p* < 0.05
	Major (*n* = 29)	10 (34.5%)	19 (65.5%)	

*p* ^∗^: Fisher's test.

## Data Availability

Data are deposited in a repository.
